# The swimming plus-maze test: a novel high-throughput model for assessment of anxiety-related behaviour in larval and juvenile zebrafish (*Danio rerio*)

**DOI:** 10.1038/s41598-018-34989-1

**Published:** 2018-11-08

**Authors:** Zoltán K. Varga, Áron Zsigmond, Diána Pejtsik, Máté Varga, Kornél Demeter, Éva Mikics, József Haller, Manó Aliczki

**Affiliations:** 1 Laboratory of Translational Behavioral Neuroscience, Department of Behavioral Neurobiology, Institute of Experimental Medicine, Hungarian Academy of Sciences, Budapest, Hungary; 2 János Szentágothai Doctoral School of Neurosciences, Semmelweis University, Budapest, Hungary; 3 Department of Genetics, Eötvös Loránd University, Budapest, Hungary; 4 Semmelweis University “Lendület” Nephrogenetic Laboratory, Hungarian Academy of Sciences, Budapest, Hungary; 5 Unit of Behavioral Studies, Institute of Experimental Medicine, Hungarian Academy of Sciences, Budapest, Hungary; 6 Laboratory of Behavioral and Stress Studies, Department of Behavioral Neurobiology, Institute of Experimental Medicine, Hungarian Academy of Sciences, Budapest, Hungary

## Abstract

Larval zebrafish (*Danio rerio*) has the potential to supplement rodent models due to the availability of resource-efficient, high-throughput screening and high-resolution imaging techniques. Although behavioural models are available in larvae, only a few can be employed to assess anxiety. Here we present the swimming plus-maze (SPM) test paradigm, a tool to assess anxiety-related avoidance of shallow water bodies in early developmental stages. The “+” shaped apparatus consists of arms of different depth, representing different levels of aversiveness similarly to the rodent elevated plus-maze. The paradigm was validated (i) in larval and juvenile zebrafish, (ii) after administration of compounds affecting anxiety and (iii) in differentially aversive experimental conditions. Furthermore, we compared the SPM with conventional “anxiety tests” of zebrafish to identify their shared characteristics. We have clarified that the preference of deeper arms is ontogenetically conserved and can be abolished by anxiolytic or enhanced by anxiogenic agents, respectively. The behavioural readout is insensitive to environmental aversiveness and is unrelated to behaviours assessed by conventional tests involving young zebrafish. Taken together, we have developed a sensitive high-throughput test allowing the assessment of anxiety-related responses of zebrafish regardless of developmental stage, granting the opportunity to combine larva-based state-of-the-art methods with detailed behavioral analysis.

## Introduction

Mental disorders, particularly those associated with anxiety represent a serious burden on both the individual and the society^1^. In order to devise therapy for such disorders, clinical and preclinical research aims to uncover the basis of anxiety-related psychopathologies and develop novel interventions. However, compounds tested on the preclinical level, despite the high resource intensity of this phase, show a low success rate in placebo controlled clinical trials^2,3^. Consequently, there is an emerging need for innovative and resource-efficient approaches that support the development of new therapeutic strategies.

The zebrafish (*Danio rerio*) is a tropical fish species^4^ rapidly gaining attention in biomedical research. The validity of this model is supported by the fact that 84% of human disease-related genes have at least one zebrafish orthologue^5^. In addition, the central nervous system of teleost fishes shares major characteristics with that of higher vertebrates, but possesses less complexity, making it eligible to model basic brain functions^6–9^. Besides these homologies, the rationale for disease modelling in zebrafish is also supported by the low maintenance costs and reduced ethical concerns associated with its use^9–11^.

A particular advantage of zebrafish models is the availability of numerous techniques to monitor and manipulate physiological processes in the larval stage. Due to the optical clarity of fish during this stage, optogenetic manipulation and imaging of central nervous system activity can be performed simultaneously, with a temporal and spatial resolution that surpasses other available models by far. Furthermore, larval fish show a wide behavioural repertoire, as early as a few days after hatching, making them an ideal model to combine behavioural and physiological screening. Despite this, relatively few approaches involve anxiety-related responses of larvae, making it difficult to conduct integrative phenomenological analysis. Furthermore, available behavioural tests utilizing larvae, e.g. the open tank (OT) and the light/dark tank (LDT) test, are based on the natural aversion of exposed^12,13^ or poorly lit areas^14,15^, but exclude aversion of the water surface, even though it is the most frequently measured, persistently expressed and reproducible defensive response to novelty in adult zebrafish^16–26^. This is possibly due to the difficulties with recording the swimming depth of fish in a high-throughput context. Designing a behavioural paradigm in larvae assessing this typical defensive response, particularly in combination with the wide range of techniques available for modifying and monitoring physiological function would allow us to investigate the basis of anxiety in a high-throughput and highly detailed manner.

In the current study, we present the swimming plus-maze (SPM) test, a paradigm developed by our group for high-throughput screening of anxiety-related behaviour in young zebrafish. The SPM platform consist of differentially deep arms, offering a choice between differently aversive zones, making it possible to analyze surface avoidance behaviour in a high-throughput manner. The test is highly analogous to the rodent elevated plus-maze (EPM) paradigm^27,28^, both in respect of its concept, design and observed pattern of behavioural outcome. To validate our test we (i.) analyzed the effects of anxiolytic and anxiogenic compounds on larvae (8 dpf) and juveniles (30 dpf), (ii.) investigated the effects of experimental conditions such as light intensity, test repetition, and the context of test batteries. Furthermore, (iii.) we aimed to determine the shared characteristics of the SPM with previous conventional tests employed in zebrafish. To the best of our knowledge, this is the first test that utilizes the anxiety-related avoidance of shallow water in zebrafish regardless of developmental stage, hence filling an important niche in larva-based high-throughput phenotypic screening.

## Results

### Exploration pattern and correlation of variables in the SPM

**Figure 1:**
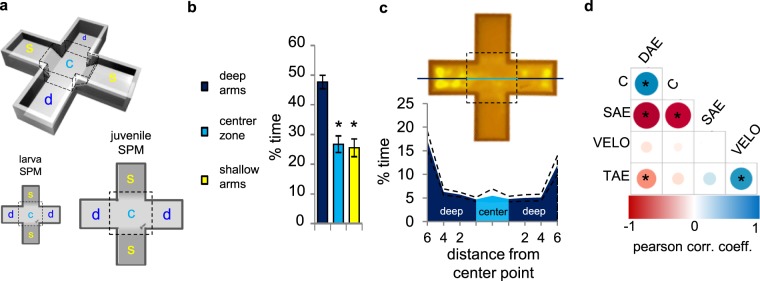
The swimming plus-maze test (SPM): apparatus, behaviour and selection of variables. (**a**) 3D (top-left) and 2D drawings of apparati for larvae (bottom-left) and juveniles (bottom-right). Dashed lines indicate the boundary of areas (d: deep arm; s: shallow arm; c: center zone (intersection + ramps)). For exact dimensions of the apparati see Table 1. (**b**) Percentage of time spent in each zone of the SPM test; (**c**) representative heatmap of exploration (top) and fine resolution analysis of deep arm activity (bottom)(dashed lines indicate mean ± SEM). (**d**) Correlation between behavioural measures of the SPM test. Size and colour intensity of dots in the intersection of variable abbreviations are in linear association with the correlation coefficient between the two variables. Bigger dot indicate stronger association. Blue dots indicate positive, red ones indicate negative correlation. *Shows significant correlation between variables. DAE: deep/total arm entries, C: choice index, SAE: shallow/total arm entries, VELO: mean velocity, TAE: total arm entries.

First, we aimed to analyze the exploration pattern (e.g. the spatial and temporal dynamics of behaviour) of zebrafish in the plus-shape platform (for dimensions see Table 1). Furthermore, we aimed to investigate the nature of connections between each measured variable to draw a general profile for the use of the SPM test. The exploration pattern and the association of variables were calculated from the data of vehicle treated, naive animals. Zebrafish prefer the deep arms over the shallow arms and the center zone (Fig. 1b) (see statistical data in Supplementary Table S1). The more detailed exploration analysis and the heatmaps revealed an emerging trend in this preference towards the outermost parts of the deep arms (Fig. 1c). According to the 1-minute-resolution analysis both larval (8 dpf) and juvenile (30 dpf) zebrafish express permanent deep arm preference throughout a 10 minute test session (Supplementary Fig. 1). Correlation analysis revealed a strong positive association between choice index and deep/total arm entries, while both of these variables negatively correlate with the relative entry frequencies to the shallow arms. Neither of the previous variables, except for total arm entries, are associated with velocity (Fig. 1d) (see statistical data in Supplementary Table S2).

**Table 1:** Dimensions of the SPM apparati for larval and juvenile fish.

Developmental stage	Zone	Length × Width	Depth	Bottom thickness
Larva (8 dpf)	deep arms	5 × 4 mm	2 mm	1 mm
	center zone (ramp + intersection + ramp)	(2 + 4 + 2) × (2 + 4 + 2) mm	2 mm	
	shallow arms	5 × 4 mm	1 mm	
Juvenile (30 dpf)	deep arms	10 × 8 mm	5 mm	
	center zone (ramp + intersection + ramp)	(3 + 8 + 3) × (3 + 8 + 3) mm	5 mm	
	shallow arms	10 × 8 mm	2.5 mm

### Pharmacological validation of the SPM

In *Experiment* 1, we sought to validate the SPM test by applying pharmacological agents affecting human anxiety.

**Figure 2:**
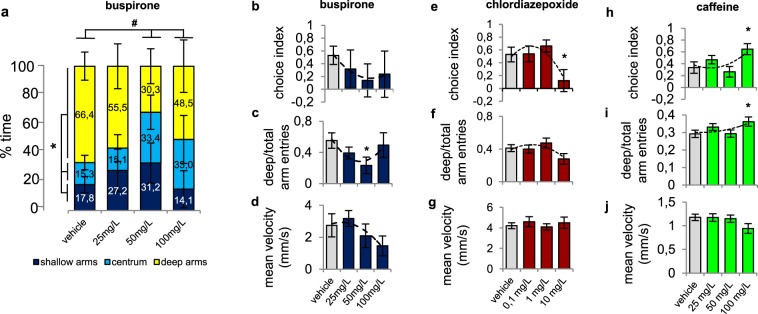
Pharmacological validation of the SPM. (**a**) Changes in spatio-temporal patterns of behaviour in response to buspirone treatment. *Significant difference from time percentage spent in deep arms, ^#^significant interaction between treatment and area. (**b**–**d**) Changes in arm preference-associated variables and velocity in response to buspirone. (**e**–**g**) Effects of chlordiazepoxide. (**h**–**j**) Effects of caffeine. *Significant difference from vehicle treated group. Dashed lines represent polynomial trendlines.

In *Experiment 1a* we assessed the effects of the anxiolytic agent buspirone on 8 dpf larva behaviour. Vehicle-treated fish have robust preference towards the deep arms over the shallow arms or the central zone. 50 mg/L buspirone decreased the time spent in the deep arms and, as the significant interaction indicates, anxiolytic treatment completely or partially abolished the control ratio (Fig. 2a) (see statistical data in Supplementary Table S1). Buspirone did not affect choice index (Fig. 2b), however, it significantly lowered deep/total arm entries compared to controls (Fig. 2c). None of the applied concentrations affected mean velocity (Fig. 2d) (see statistical data in Table 2).

**Table 2:** Statistical data of the pharmacological validation experiments (*Experiment 1a*, *1b*, and *1c*) shown in Fig. 2b–j.

Experiment	Compared groups	Measure	Estimate	SE	*n*	*t-*value	*p*-value
1a (buspirone)	vehicle *vs* 25 mg/L	choice index	0.31	0.36	8 *vs* 10	0.88	0.358
		deep/total arm entries	−0.17	0.15		−1.117	0.273
		mean velocity	0.69	0.89		0.77	0.448
	vehicle *vs* 50 mg/L	choice index	0.42	0.37	8 *vs* 10	1.12	0.182
		deep/total arm entries	−0.32	0.15		−2.055	0.049*
		mean velocity	0.39	0.90		−0.43	0.670
	vehicle *vs* 100 mg/L	choice index	0.25	0.38	8 *vs* 10	0.68	0.500
		deep/total arm entries	−0.05	0.16		−0.293	0.772
		mean velocity	−1.11	0.92		−1.21	0.237
1b (chlordiazepoxide)	vehicle *vs* 0.1 mg/L	choice index	0.17	0.19	25 *vs* 15	0.906	0.390
		deep/total arm entries	−0.02	0.07		−0.234	0.816
		mean velocity	0.28	0.53		0.525	0.612
	vehicle *vs* 1 mg/L	choice index	0.01	0.15	25 *vs* 15	0.098	0.922
		deep/total arm entries	0.06	0.07		0.842	0.402
		mean velocity	0.28	0.46		0.620	0.546
	vehicle *vs* 10 mg/L	choice index	0.53	0.18	25 *vs* 16	2.870	0.024*
		deep/total arm entries	−0.14	0.07		−1.922	0.059
		mean velocity	0.46	0.52		0.887	0.391
1c (caffeine)	vehicle *vs* 25 mg/L	choice index	−0.13	0.12	26 *vs* 17	−1.016	0.313
		deep/total arm entries	0.04	0.03		1.383	0.171
		mean velocity	−0.004	0.11		−0.039	0.969
	vehicle *vs* 50 mg/L	choice index	0.06	0.12	26 *vs* 19	0.490	0.625
		deep/total arm entries	0.01	0.03		0.459	0.648
		mean velocity	−0.03	0.11		−0.271	0.787
	vehicle *vs* 100 mg/L	choice index	−0.32	0.13	26 *vs* 15	−2.401	0.019*
		deep/total arm entries	0.07	0.03		2.308	0.024*
		mean velocity	−0.22	0.12		−1.862	0.066

We set treatment “vehicle” as reference levels in each linear mixed model. *P*-values in red with asterix represent significant difference from vehicle treated group. Values in orange represent marginal significance.

In *Experiment 1b* we investigated the effects of the anxiolytic agent chlordiazepoxide on 8 dpf larval behaviour. Vehicle-treated fish showed a highly positive choice index towards the deep arms, which was significantly lowered by 10 mg/L chlordiazepoxide (Fig. 2e). Chlordiazepoxide only marginally decreased deep/total arm entries (Fig. 2f) and did not affect mean velocity (Fig. 2g) (see statistical data in Table 2).

In *Experiment 1c* we assessed the effects of the anxiogenic compound caffeine on 8 dpf larva behaviour. Control zebrafish showed strong deep arm activity, which was increased by the highest concentration of caffeine, as indicated by the enhanced choice index (Fig. 2h) and deep/total arm entries (Fig. 2i). Also, caffeine caused a marginal decrease in velocity (Fig. 2j) (see statistical data in Table 2).

### Validation of the SPM in juvenile zebrafish

**Figure 3:**
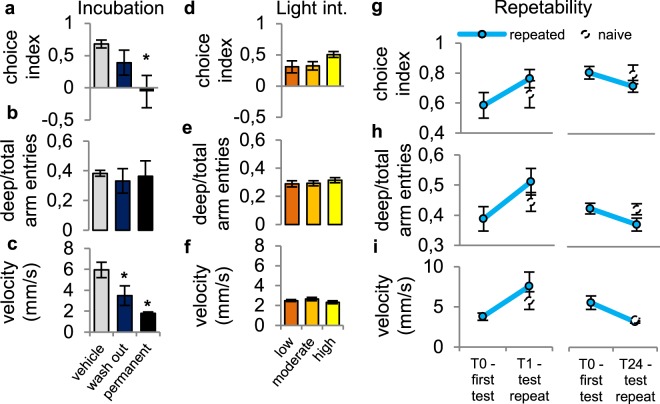
Validation in juveniles and the effect of environmental aversiveness and familiarity. (**a**–**c**) Behaviour of juvenile zebrafish after buspirone treatment. (**e**–**g**) Effects of different light intensities on juvenile behaviour. (**h**–**j**) Effects of test repetition on juvenile behaviour. T0: results from the first test of repeated groups, T1 and T24: results from the tests of naive groups and the second tests of repeated groups after 1 and 24 hour intervals.

In *Experiment 2*, we sought to validate the SPM test in juvenile zebrafish, hence we treated 30 dpf fish with 50 mg/L buspirone, which was shown to be effective in the case of larvae, applying different incubation protocols (washout vs. continuous treatment). Continuous exposure to buspirone significantly lowered deep arm preference (Fig. 3a) but not deep/total arm entries (Fig. 3b). However, both treatments decreased mean velocity (Fig. 3c) (see statistical data in Table 3).

**Table 3:** Statistical data of the validation in juvenile fish (*Experiment 2*) shown in Fig. 3a–c.

Experiment	Compared groups	Measure	Estimate	SE	*n*	*t-*value	*p*-value
2 (buspirone on juvenile behaviour)	vehicle *vs* wash-out	choice index	0.29	0.25	14 *vs* 11	1.172	0.250
		deep/total arm entries	−0.07	0.10		−0.774	0.445
		mean velocity	−2.42	0.98		−2.462	0.020*
	vehicle *vs* permanent	choice index	0.74	0.24	14 *vs* 12	3.036	0.005*
		deep/total arm entries	−0.04	0.09		−0.465	0.646
		mean velocity	−3.92	0.94		−4.147	0.0003*

We set treatment “vehicle” as reference level in the fitted linear mixed model. *P*-values in red with asterix represent significant difference from vehicle treated group. Values in orange represent marginal significance.

### Effects of environmental aversiveness on behaviour in the SPM

In *Experiment* 3, we aimed to investigate the sensitivity of the SPM to testing conditions such as environmental aversiveness, hence we exposed 30 dpf juvenile fish to different light intensities during testing. Results from the low-light-exposed group were set as the reference level of the model. Neither moderate nor intense illumination affected the measured behavioural variables compared to the low light intensity group (Fig. 3d–f) (see statistical data in Supplementary Table S3).

### Effects of repeated testing in the SPM

In *Experiment 4a* and *4b* we also aimed to assess the sensitivity of the SPM to environmental conditions such as repeated exposure to the test, hence we tested 30 dpf juvenile zebrafish repeatedly in 1 and 24 hour intervals, respectively, using additional naïve control animals in the second sampling. Results from the second test were set as the reference level of the model. Behaviour measured in the repeated test did not differ from baseline values or from the results of naïve controls (Fig. 3g–i) (see statistical data in Supplementary Table S3).

### Shared characteristics of the SPM with conventional anxiety tests

**Figure 4:**
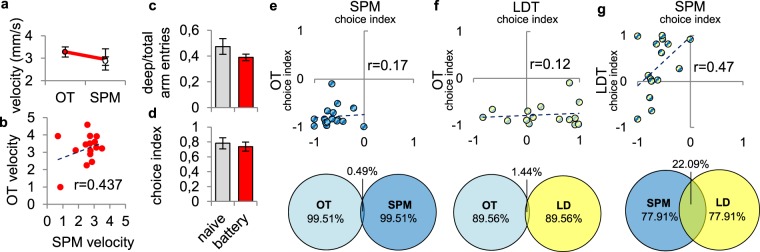
Shared characteristics of SPM and conventional “anxiety tests” of zebrafish. (**a**) Mean velocity in the OT and in the SPM tests of double tested (red) and naïve (white) animals. (**b**) Intra-individual correlation of velocity measured in the SPM and OT tests. (**c**,**d**) Deep arm activity related variables measured in naïve and battery tested (prior OT exposure) subjects. (**e**–**g**) Correlation plots and venn-diagrams of association between domains of avoidance behavior measured by different “anxiety tests” of zebrafish (OT: open tank, LDT: light/dark tank, SPM: swimming plus maze). The magnitude of avoidance is represented as choice indices. Venn-diagrams show percentage of shared variance between tests, calculated as r^2^ * 100, where r is the correlation coefficient between choice indices.

In *Experiment* 5 we sought to unravel shared characteristics of behavioral domains measured by the SPM and conventional zebrafish anxiety tests by comparing primary outcomes that describe avoidance behavior in these paradigms. We subjected 30 dpf juvenile zebrafish to the open tank (OT), SPM, and light/dark tank (LDT) tests in rapid succession. In order to assess whether behaviour in the SPM is affected by prior OT testing, we introduced an additional parallel-tested control group, naïve to any test exposure. Choice index, deep/total arm entries, and mean velocity were unaffected by previous OT exposure compared to test naïve controls (Fig. 4c,d) (see statistical data in Supplementary Table S3). Surprisingly, mean velocity in the SPM and OT tests did not correlate significantly (Fig. 4b). Also, there was no significant correlation between the choice indices of SPM and OT (Fig. 4e) or SPM and LD (Fig. 4g). However, according to the correlation coefficients between these variables, SPM and LD shared 22.9% of variance, in contrast to the SPM-OT comparison, in which this measure was only 0.49% (see statistical data in Supplementary Table S4).

## Discussion

Our results show that both larval and juvenile zebrafish prefer the deep arms over the shallow arms or the central zone. This preference was abolished by the clinical anxiolytics buspirone and chlordiazepoxide, while it was enhanced by the anxiogenic caffeine. The SPM was applicable regardless of the developmental stage of the behavioural phenotype, the aversiveness or the familiarity of the testing environment, or prior OT exposure. Interestingly, deep arm activity in the SPM was unrelated to behavioural responses measured by conventional “anxiety tests” of larval zebrafish.

The primary behavioural responses in our study aimed to describe arm preference and the drive behind it. Choice index and deep/total arm entries are strongly correlated variables, however, neither of these are associated with the locomotion of zebrafish, suggesting that these variables are associated with closely related, if not the very same motivational states, and thus are potentially substitutable with each other. This relationship indicates an important difference between the SPM and EPM, since in the latter test, the relative entries into one of the less aversive arms are a predictor of locomotion but not anxiety^28^. In contrast, in the SPM locomotion is predicted only by the number of total arm entries, according to its high correlation with the average velocity of larvae. Despite the fact that in the rodent analogue of the SPM the number of arm entries is widely accepted as a measure of locomotion^28^, we suggest the use of more than one variable due to some discrepancies in the measurement of locomotion reported in fish by Ingebretson and Masino^29^. According to their study, smaller distance moved and slower velocity of fish is in some cases accompanied by more active swimming episodes, a phenomenon that did not occur in rodent experiments. However, different measures of activity in the more aversive zone, e.g. time spent and entry frequencies into the open arm are both loaded on the same factor in the principal component analysis of EPM variables^30^. This finding is in line with the strong correlation between such measures in the SPM test, supporting the similarity of the two paradigms.

To the best of our knowledge, our group is the first that has observed and utilized preference towards areas where deeper water is accessible in larval zebrafish. Such marked preference for these zones can possibly be based on the bottom-dwelling geotactic behaviour of zebrafish shown in response to novelty, described in adults by several groups using either the novel tank diving test or a plus maze with a ramp^18,21,26^. This similarity is supported by the fact that in our experiments both 8 dpf larvae and 30 dpf post-metamorphic juveniles, showing a completely developed behavioural phenotype^31,32^, expressed similar behavioural patterns regarding arm preference. There is a growing body of evidence in adult zebrafish that this phenomenon is driven by the aversion to the water surface rather than preference for the bottom^33^ and that direct exposure to such stimuli is highly stressful for fish^34,35^. Blaser and Goldsteinholm constructed a two-sided platform for adult fish with a visual cliff apparatus, in which the distance from the water surface and bottom could be manipulated independently. In one condition they equalized water depth in the two sides using a glass insert, but the visual depth was manipulated by placing a gravel substrate just below, or far below the glass floor. Zebrafish preferred the side that looked deeper and not the one that looked closer to the gravel, indicating that escape from the surface, rather than approach to the bottom motivates diving behavior. Furthermore, Ingebretson and Masino found that larval zebrafish show significantly less active swimming in shallower bodies of water, also, a higher ratio of zebrafish stayed completely immobile in such conditions^29^. This response can possibly be interpreted as an increase in anxiety-related freezing behaviour of larvae. The nature of the relationship between the relative depth of water and the level of aversiveness in larval stage is going to be the aim of future investigations. Interestingly, while the central zone of the SPM is as equally deep as the deep arms, this area was less attractive to zebrafish in our study, suggesting that the intersection of the platform represents a different, possibly more aversive stimulus, e.g. higher environmental exposure. These results indicate that the exploration pattern in the SPM is driven by more than one stimulus and the associated internal states. Importantly, possible differences of light intensity in the deep and shallow arms stemming from the varying distances to the light source are very unlikely to lead to deep arm preference, as exploration patterns were unaffected by different light conditions. This is also supported by the fact that larval and post-metamorphic fish showed very similar behaviour in the SPM, despite a switch occurring from a light preferring to a dark preferring phenotype between these developmental stages^32^. Taken together, the observed behaviour is possibly the result of a trade-off between highly exposed aspects of the SPM, e.g. water surface or the intersection, and the innate urge of animals to explore the novel environment.

In our pharmacological validation experiments, deep arm activity was decreased by anxiolytics buspirone and chlordiazepoxide, while it was increased by the axiogenic caffeine, without either affecting locomotion. Since earlier reports on the effects of buspirone and benzodiazepines are rather inconsistent^36–40^, it is an important feature of the SPM that both agents, regardless of their target of action, affected behaviour in a similar manner. Furthermore, another advantage of the SPM is that, contrary to other tests^13^, there was no measurable ceiling-effect potentially masking the anxiogenic properties of caffeine. Interestingly, the employed anxiolytics affected different, although strongly correlated variables, pointing out the importance of detailed behavioural analysis. It is also important to note that, in contrast to earlier reports^41^, we did not detect the sedative effects of chlordiazepoxide. This phenomenon might be attributed to a sum of several effects, e.g. strain differences or lower sensitivity of the SPM for measuring motor changes. Despite this small limitation, our findings suggest that the basis of behaviour shown in the SPM is anxiety-related.

Our experiment in juveniles revealed that the behavioural patterns of vehicle-treated juvenile zebrafish are very similar to those measured in larvae. The significance of applying such an experiment to 30 dpf fish is that most behavioural domains, e.g. social or light/dark avoidance behaviour, have already developed to their mature form by this age^31,32^. Based on our analysis, including fish from different developmental stages, and the investigation of adult behaviour by other labs^18,21,26^, it is very likely that surface avoidance behaviour remains conserved throughout the ontogenesis. Such phenomena enable the consistent use of SPM, regardless of developmental stage, over other current methods. However, 50 mg/L buspirone, previously shown to be effective in larvae, affected deep arm activity only in the case of the longer incubation protocol, but decreased locomotion in all treatment groups. This divergent behavioural pattern was probably rather due to the maturation of the CNS than differing ability to absorb the compound, since the treatment exerted sedative effect in every case. Revealing the basis of these surprising effects is going to be the aim of future investigations, however, our results suggest that SPM is applicable in different developmental stages, thus enabling it to be used for behavioural analysis during the early stages of ontogenesis.

In our experiments aiming to determine the effects of environmental conditions, neither different light intensities, nor repeated testing influenced the behaviour of juvenile fish. However, there is a non-significant trend in the test repetition experiment suggesting an enhanced excitation after the 1 hour and a mild habituation after the 24 hours intervals. These results suggest that these aspects of aversiveness or familiarity of the testing environment do not act as a strong biasing factors in the SPM, in contrast to its rodent analogue^27,28,42,43^. Moreover, the use of naive controls revealed that there is no considerable fluctuation in the behavioural patterns in 1 or 24 h intervals. Our results suggest that the SPM paradigm can be employed in a flexible manner even in a high-throughput context.

To determine whether deep arm activity in the SPM is related to other forms of avoidance behaviour measured by conventional “anxiety tests” of zebrafish, e.g. OT and LDT, we compared these. While the behavioural endpoints of the different paradigms only show a slight correlation, avoidance behaviour of juvenile fish in the SPM and LDT shared 22.9% variance, reflecting much more similarity between these tests than between their rodent counterparts^44^. However, such weak associations between behavioural measures seem counterintuitive considering that each test relies on unconditioned avoidance of threatening situations and measures behaviours that are theoretically based on the same emotional construct. According to the rodent literature, such discrepancies are often attributed to differences between correlation analysis of scores of individuals versus means of treatment groups^45^. Ramos stated that despite the fact that rodent open field, light/dark box and EPM tests are all sensitive to anxiolytic drugs, the baseline behaviour of untreated animals might not present significant inter-test correlations. He explained the latter phenomenon as correlating behaviour of individuals burdened by inter-individual variability, such as the transient condition, e.g. anxiety state, of the animals, that can be important to the point of overriding the anxiety trait of a given group of animals. Consequently, similarly to rodent studies, meta-analysis of anxiety tests provide more valuable data about the relatedness of different paradigms. Kysil and colleagues made the most comprehensive comparative analysis of the LDT and the novel tank diving tests so far^20^, and found that these test have a good cross-test validity and similar sensitivity to zebrafish anxiety-like states. In contrast, detailed studies based upon direct comparison of the tests^23^ or their major stimuli, e.g. water depth and wall colour^18^, found several differences in the background drive of the expressed behaviour, emphasizing the importance of using multiple tests for phenotyping zebrafish. This data supports the idea that emotionality is not unidimensional, but varies along several partially overlapping axes that are only accessible through a series of tests. Kysil also describes that the context of the novel tank diving protocol exerts higher cortisol response and a more survival-driven innate diving behaviour indicating a more stressful testing environment for fish^20^. In our experiments, in line with such findings, zebrafish expressed much stronger avoidance in the OT and SPM, than in the LDT test. Considering the data above and our own results, i.e. (i.) OT exposure did not influence subsequent SPM behaviour, (ii.) all three tests applied measure different phenomenological correlates of anxiety and (iii.) are differentially sensitive to motor changes, we recommend the use of the test battery applied here to analyze behavioural phenotypes in detail.

In summary, we have determined that larval and juvenile zebrafish show a yet unobserved behavioural pattern, which is possibly based on bottom-dwelling behaviour, exerted by complex stimuli of the SPM test. This pattern can be manipulated by pharmacological agents similarly to anxiety-based responses of higher vertebrates, including humans. With the employment of the SPM paradigm we are able to screen genotypes, adverse experiences or any type of manipulations potentially affecting anxiety in a more detailed and highly efficient manner due to the utilization of larvae instead of adults. In addition, with the use of SPM, we are potentially able to combine state-of-the-art methods, based on the unique advantages of young zebrafish, e.g. *in vivo* imaging techniques, with detailed behavioural analysis offered by the presented test battery.

## Materials and Methods

### Animals

Wild type (AB) fish lines were maintained in the animal facility of ELTE Eötvös Loránd University according to standard protocols (Westerfield, 2000). Experimental subjects were unsexed animals aged 8 or 30 dpf (days post fertilization). Fish were group-housed and maintained in a standard 14 h/10 h light/dark cycle. Animals were terminated on ice immediately after each experiment. Feeding of larvae started at 5 dpf with commercially available dry food (a 1:1 combination of <100 μm and 100–200 μm Zebrafeed, Sparos) combined with paramecium. This regimen lasted until 15dpf, after that juvenile fish were fed using dry food with gradually increasing particle size (200–400 μm Zebrafeed) combined with fresh brineshrimp hatched in the facility. From 30 dpf adult fish were fed with dry food (Small Granular, Special Diets Services, product code: 824876) combined with brine shrimp. Water quality was controlled constantly by a Stand-Alone (Tecniplast) system, with parameters set at pH = 8.0, conductivity = 500 µS, temperature = 28 °C. Animal density never exceeded. All protocols employed in our study were approved by the Hungarian National Food Chain Safety Office (Permits Number: PEI/001/1458-10/2015 and PE/EA/2483-6/2016).

### Drugs

All compounds were dissolved in E3 medium and administered as a water bath followed by a brief washout (except when testing was conducted in the treatment solution) in compartments of a 24-well plate. Each compartment (diameter = 15.6 mm) contained 1.5 ml of treatment solution. We applied buspirone (PubChem CID: 24278079) and caffeine (PubChem CID: 2519) at the concentrations of 0 (vehicle), 25, 50, and 100 mg/l, and chlordiazepoxide (PubChem CID: 24892497) at the concentrations of 0 (vehicle), 0.1, 1, and 10 mg/l. Concentrations were selected based on earlier studies^19,41^. Compounds were obtained from Sigma-Aldrich.

### Behavioural tests and analysis

#### The SPM test

The swimming plus-maze (SPM) apparatus is a “+” shaped platform consisting of 2 + 2 opposite arms, different in depth, connected by a center zone. The platforms were 3D printed by a Stratasys Objet30 printer using white opaque PolyJet resin. To test both larvae and juveniles we designed two types of apparatuses matching the sizes of the subjects (for specifications see Table 1). It is important to note that the center zone and deep arms had the same depth, however, exploration patterns (Video S1) shown by the detailed exploration analysis (Fig. 1c) and the heatmap of exploration (Fig. 1d) suggest that individuals actively discriminate these areas. Shallow arms did not act as physical barriers of fish movement in either type of platforms (Videos S2 and S3).

Experiments were conducted during the second part of the light phase, as zebrafish have been reported to show continuous activity in this period^46^. During a single trial, 8–16 subjects were tested simultaneously. The orientation of the platforms were randomized. Experiments in which pharmacological treatments were employed began with a 10 minute treatment bath in a 24-well plate compartment after which animals were gently pipetted into another compartment containing E3 medium for a brief washout (except when testing was conducted in the treatment solution) then to the SPM for 5 or 10 minutes and recorded by a video camera. The order of treatments was sorted by using a sequence of random numbers generated by the RAND function of MS Excel. The experimenter who pipetted the animals was blind to treatment groups. Experiments were carried out in an examination chamber and illuminated from beneath with white LED panels covered by matte Plexiglass. To reduce interfering stimuli from the environment the unit was covered with a black plastic box with a hole on top allowing the attachment of a video camera. Video recordings were analyzed with EthoVision XT 12 automated tracker software^47^. The experimenter who conducted the analysis was blind to treatment groups until the sortation process of analyzed data.

Time spent in each zone and the mean of overall velocity was measured. To characterize arm preference, a choice index was defined as the relative time spent in the deep compared to in shallow arms. Consequently, a choice index of 1 indicates 100% time in deep arms, whereas a choice index of −1 represents 100% time in the presumably more aversive shallow arms.

$$\frac{time\,spent\,in\,deep\,arms}{time\,spent\,in\,deep+shallow\,arms}-\frac{time\,spent\,in\,shallow\,arms}{time\,spent\,in\,deep+shallow\,arms}\,=-\,{1}\le choice\,index\le +\,{1}$$

Deep/total arm entries in SPM describes the relative frequency of the cases when an animal enters one of the deep arms. To conduct detailed exploration analysis of the deep arms and the center zone we measured the time spent in 3 equal complementary sections in each zones and calculated the average slope of these.

#### The OT test

For open tank (OT) testing, standard, completely transparent 24-well plates (d = 15.6 mm) were used.

10-minute-long behavioural tests were conducted using the same protocol as in the case of SPM.

To describe thigmotaxis (edge preference) a choice index was defined as the relative time spent in the outer 20% of the compartment compared to time spent in the center zone.

$$\frac{time\,spent\,in\,periphery}{total\,time}-\frac{time\,spent\,in\,centrum}{total\,time}=-\,{1}\le choice\,index\le +\,{1}$$

Mean of overall velocity was also measured.

#### The LDT test

For light/dark tank (LDT) testing, half of the compartments of a standard 24-well plate were masked with opaque matte black paint.

10-minute-long behavioural tests were conducted using the same protocol as in the case of OT and SPM.

To describe scotophobia (dark avoidance) a choice index was defined as the relative time spent in the dark zone of the compartment compared to time spent in the light zone.

$$\frac{time\,spent\,in\,dark\,zone}{total\,time}-\frac{time\,spent\,in\,light\,zone}{total\,time}=-\,{1}\le choice\,index\le +\,{1}$$

### Experimental design

#### Pharmacological validation of the SPM

In *Experiment 1a*, *1b*, and *1c*, we validated the SPM paradigm by the administration of pharmacological agents shown to affect anxiety in preclinical rodent models and human clinical trials as well. Different sets of 8 dpf larvae were acutely treated with either the anxiolytics buspirone (0, 25, 50 and 100 mg/L) (*Experiment 1a*), chlordiazepoxide (0, 0.1, 1, and 10 mg/L) (*Experiment 1b*) or the anxiogenic caffeine (0, 25, 50 and 100 mg/L) (*Experiment 1c*) and their behaviour was recorded for 5 minutes. Sample sizes were 8–10 per group in *Experiment 1a*, 15–25 per group in *Experiment 1b*, and 15–26 per group in *Experiment 1c*.

#### Validation of the SPM in juvenile zebrafish

As juvenile, post-metamorphic fish avoid different stimuli than larvae, e.g. prefer dark over brightly lit environments^32^, in *Experiment 2a* and *2b*, we determined the validity of the SPM paradigm for juvenile zebrafish. 30 dpf fish were acutely treated with 50 mg/L of buspirone, the dose shown to be effective in larvae, and their behaviour was recorded for 5 minutes in the apparatus designed for juveniles. Since juvenile fish, due to their less permeable skin, absorb compounds differently than larvae^48,49^, we introduced an additional group which we placed into the testing apparatus in their treatment solution, providing longer incubation times (15 minutes). Sample sizes were 11–14 per group.

#### Effects of environmental aversiveness on behaviour in the SPM

In *Experiment* 3 we assessed the effects of environmental aversiveness on behaviour in the SPM. 30 dpf juveniles were tested under different light intensities representing different levels of environmental aversiveness^28^, and their behaviour was monitored for 10 minutes. As light conditions were set by the examination chamber lighting for all platforms in a particular trial, subjects from similar treatment groups were tested in one trial. The order of the trials was randomized. Sample sizes were 16 animals in each group.

#### Effects of repeated testing in the SPM

In *Experiment* 4, we aimed to investigate the reproducibility of the SPM test. 30 dpf juvenile zebrafish were tested two times in 1 or 24 hour intervals and their behaviour was recorded for 10 minutes. In both cases, we tested a naïve control group as well in the second sampling period. Sample sizes were 10 in each group.

#### Shared characteristic of the SPM with conventional anxiety tests

In *Experiment* 5, we aimed to determine the shared characteristics of the SPM with other tests measuring anxiety-like behaviour in larval zebrafish and its suitability for use in test batteries. 30 dpf juvenile zebrafish were tested for 10 minutes in the OT, SPM and then the LDT test. In the case of SPM, we introduced an additional control group, without preliminary OT testing, to determine the effects of prior novelty exposure on SPM behaviour too.

### Data analysis

Data is represented as mean ± SEM. We performed statistical analysis using R Statistical Environment^50^. To analyze the effects of pharmacological agents or experimental conditions on behavioural variables we used linear mixed models^51^ from the *lme4* package^52^. To separate variance stemming from time or sequence of experimental trials or location of test platforms, these factors were added as random effects to our models. To analyze within-group differences between percentage of time spent in each zone, we fitted linear mixed models with zone*treatment interaction as fixed, and subject identifiers as random effects. We set treatment “vehicle” and zone “deep arms” as reference levels. To determine correlation between SPM variables and also between measures of different anxiety tests, we computed Pearson correlation coefficients (*r*) using the *GGally* package^53^. To create venn-diagrams of these correlations, we calculated percentage of shared variance applying the *r*^2^ × 100 formula, where *r* is the correlation coefficient. We calculated *p*-values from *t*-values of *lme4* using *lmerTest* package^54^ and rejected H_0_ if *p*-values were lower than 0.05.

## Electronic supplementary material

supplementary information

centrum exploration of larval zebrafish

larval zebrafish in the SPM (8 dpf)

juvenile zebrafish in the SPM (30 dpf)

## Data Availability

The datasets generated during the current study are available in the *figshare* repository (https://figshare.com/s/0ec47f2ffbe73f35500d).
