# Supplementary material for: The swimming plus-maze test: a novel high-throughput model for assessment of anxiety-related behaviour in larval and juvenile zebrafish (Danio rerio)
Source: Sci Rep. 2018 Nov 8;8:16590. doi: 10.1038/s41598-018-34989-1 (PMC6224510; doi:10.1038/s41598-018-34989-1)
Supplement: Supplementary file 1 — supplementary information [file 41598_2018_34989_MOESM1_ESM.docx]

**Supplementary information**

**Title:** The swimming plus-maze test: a novel high-throughput model for assessment of anxiety-related behaviour in larval and juvenile zebrafish (*Danio rerio*).

**Authors:** Zoltán K Varga^1,2^, M.Sc.; Áron Zsigmond^3^, Ph.D.; Diána Pejtsik^1^; Máté Varga^3,4^, Ph.D.; Kornél Demeter^5^, Ph.D.; Éva Mikics^1^, Ph.D.; József Haller^6^, D.Sc.; Manó Aliczki^1*^, Ph.D.

**Affiliations: 1** Laboratory of Translational Behavioral Neuroscience, Department of Behavioral Neurobiology, Institute of Experimental Medicine, Hungarian Academy of Sciences, Budapest, Hungary; **2** János Szentágothai Doctoral School of Neurosciences, Semmelweis University, Budapest, Hungary; **3** Department of Genetics, Eötvös Loránd University, Budapest, Hungary; **4** Semmelweis University "Lendület" Nephrogenetic Laboratory, Hungarian Academy of Sciences, Budapest, Hungary; **5** Unit of Behavioral Studies, Institute of Experimental Medicine, Hungarian Academy of Sciences, Budapest, Hungary; **6** Laboratory of Behavioral and Stress Studies, Department of Behavioral Neurobiology, Institute of Experimental Medicine, Hungarian Academy of Sciences, Budapest, Hungary

**Corresponding author:** Manó Aliczki, PhD; Laboratory of Translational Behavioral Neuroscience, Department of Behavioral Neurobiology, Institute of Experimental Medicine, Hungarian Academy of Sciences, Szigony u. 43, 1083, Budapest, Hungary; email: aliczki.mano@koki.mta.hu**Figure S1**

**Time dynamics of deep arm activity of larval and juvenile fish in a 10 minute test session.** Choice indices are presented relative to the first timebin. Dashed lines indicate ±SEM of choice index measured in the first timebin.

**Table S1**

| Expeiment | level of analysis | zone | estimate | SE | *n* | *t*-value | *p*-value |
| --- | --- | --- | --- | --- | --- | --- | --- |
| Exploration pattern | vehicle treated | deep arms *vs* centrum | -20.96 | 3.83 | 26 | -5.47 | 1.89e-06* |
|  |  | deep *vs* shallow arms | -22.18 | 3.83 |  | -5.79 | 6.42e-07* |
| 1a (buspirone) | Reference  (vehicle treated) | deep arms *vs* centrum | -66.63 | 18.91 | 8 | -3.52 | 0.002* |
|  |  | deep *vs* shallow arms | -55.73 | 19.77 |  | -2.82 | 0.009* |
|  | Interaction  (25 mg/L buspirone*zone) | deep arms *vs* centrum | 29.67 | 22.72 | 10 | 1.306 | 0.196 |
|  |  | deep *vs* shallow arms | 35.88 | 23.21 |  | 1.546 | 0.126 |
|  | Interaction  (50 mg/L buspirone*zone) | deep arms *vs* centrum | 50.22 | 23.20 | 10 | 2.16 | 0.033* |
|  |  | deep *vs* shallow arms | 58.72 | 23.6 |  | 2.49 | 0.015* |
|  | Interaction  (100 mg/L buspirone*zone) | deep arms *vs* centrum | 50.22 | 23.20 | 10 | 2.164 | 0.033* |
|  |  | deep *vs* shallow arms | 13.10 | 23.59 |  | 0.556 | 0.580 |

**Statistical data of spatio-temporal analysis of *Experiment 1* shown in figure 2a.** To analyze within-group differences between percentage of time spent in each zone, we fitted linear mixed models with zone*treatment interaction as fixed, and subject identifiers as random effects. We set treatment “vehicle” and zone “deep arms” as reference levels. Significant interaction in treatment levels, e.g. “Interaction (50 mg/L buspirone*zone)”, means that the relation between times spent in the analyzed zone (centum or shallow arms) and the reference zone (deep arms) is significantly differ from the relation between time spent in such zones at the reference treatment level e.g. “Reference (vehicle treated)”. Estimates are represented relative to reference levels. * means significant differences.

**Table S2**

|  | DAE | C | SAE | VELO | TAE |  |  |
| --- | --- | --- | --- | --- | --- | --- | --- |
| DAE | **1** |  |  |  |  |  |  |
| C | **0.65**  2.003e-08* | **1** |  |  |  |  |  |
| SAE | **-0.83**  2.272e-16* | **-0.79**  2.476e-14* | **1** |  |  |  |  |
| VELO | **-0.14**  0.267 | **-0.08**  0.522 | **-0.003**  0.9816 | **1** |  |  |  |
| TAE | **-0.45**  0.00032* | **-0.16**  0.2207 | **0.19**  0.1456 | **0.58**  1.019e-06* | **1** |  |  |

**Statistical data of correlation analysis between SPM variables shown in figure 1d.** Bold values in the intersection of variables represent pearson correlation coefficients. Values in the bottom of cells represents *p*-values. DAE: deep/total arm entries, C: choice index, SAE: shallow/total arm entries, VELO: mean velocity, TAE: total arm entries. * means signficant correlation.

**Table S3**

| Experiment ID | compared groups | measure | estimate | SE | *n* | *t-*value | *p*-value |
| --- | --- | --- | --- | --- | --- | --- | --- |
| 3 (light intensities) | low *vs* moderate light | choice index | 0.016 | 0.10 | 16 *vs* 16 | -0.16 | 0.876 |
|  |  | deep/total arm entries | 0.004 | 0.03 |  | 0.15 | 0.879 |
|  |  | mean velocity | 0.16 | 0.19 |  | 0.84 | 0.408 |
|  | low *vs* intense light | choice index | 0.20 | 0.10 | 16 *vs* 16 | -1.89 | 0.066 |
|  |  | deep/total arm entries | 0.03 | 0.03 |  | 0.94 | 0.355 |
|  |  | mean velocity | -0.18 | 0.19 |  | -0.95 | 0.351 |
| 4a (1 hour interval) | baseline *vs* repeated | choice index | 0.19 | 0.14 | 9 *vs* 9 | 1.31 | 0.239 |
|  |  | deep/total arm entries | 0.12 | 0.06 |  | -1.93 | 0.111 |
|  |  | mean velocity | 5.16 | 3.73 |  | -1.38 | 0.235 |
|  | repeated *vs* naïve | choice index | 0.15 | 0.11 | 9 *vs* 9 | 1.36 | 0.184 |
|  |  | deep/total arm entries | 0.05 | 0.05 |  | -0.1 | 0.328 |
|  |  | mean velocity | 1.96 | 1.10 |  | -0.98 | 0.338 |
| 4b (24 hour interval) | baseline *vs* repeated | choice index | 0.09 | 0.08 | 12 *vs* 12 | -1.18 | 0.285 |
|  |  | deep/total arm entries | 0.05 | 0.03 |  | 1.64 | 0.141 |
|  |  | mean velocity | 2.46 | 1.62 |  | 1.52 | 0.179 |
|  | repeated *vs* naïve | choice index | 0.08 | 0.07 | 12 *vs* 16 | -1.13 | 0.269 |
|  |  | deep/total arm entries | 0.05 | 0.03 |  | 1.95 | 0.060 |
|  |  | mean velocity | 0.34 | 0.28 |  | 1.19 | 0.241 |
| 5 (test battery) | SPM naïve *vs* SPM repeated | choice index | 0.12 | 0.09 | 16 vs 16 | -1.31 | 0.202 |
|  |  | deep/total arm entries | -0.08 | 0.07 |  | -1.3 | 0.205 |
|  |  | mean velocity | -0.07 | 0.45 |  | -0.16 | 0.873 |
|  | OT naïve *vs* SPM repeated | choice index | - | - | 16 vs 16 | - | - |
|  |  | deep/total arm entries | - | - |  | - | - |
|  |  | mean velocity | 0.33 | 0.38 |  | 0.87 | 0.393 |

**Statistical data of *Experiment 3, 4a,* *4b and 5* shown in figure 3d-3i and figure 4a-4d.** We set treatment “low light” (*Experiment 3*), test “repeated” (*Experiment 4*) and condition “battery” (*Experiment 5*) as reference levels in the fitted linear mixed models.

**Table S4**

|  | OT | SPM | LDT |
| --- | --- | --- | --- |
| OT | **1** |  |  |
| SPM | **0.07**  0.794 | **1** |  |
| LDT | **0.12**  0.670 | **0.47**  0.076 | **1** |

**Statistical data of correlation analysis between “anxiety tests” of zebrafish shown in figure 4e-4g.** Bold values in the intersection of variables represent pearson correlation coefficients. Values in the bottom of cells represents *p*-values. OT: open tank test, SPM: swimming plus-maze test, LDT: light/dark tank test * means signficant correlation.
